# Identification of an enhancer region within the *TP63*/*LEPREL1* locus containing genetic variants associated with bladder cancer risk

**DOI:** 10.1007/s13402-018-0393-5

**Published:** 2018-06-28

**Authors:** Aleksandra M. Dudek, Sita H. Vermeulen, Dimitar Kolev, Anne J. Grotenhuis, Lambertus A. L. M. Kiemeney, Gerald W. Verhaegh

**Affiliations:** 1 Department of Urology, Radboud Institute for Molecular Life Sciences, Radboud University Medical Center, Geert Grooteplein Zuid 28, 6525 GA Nijmegen, The Netherlands; 2 Department for Health Evidence, Radboud Institute for Health Sciences, Radboud University Medical Center, Nijmegen, The Netherlands

**Keywords:** Bladder cancer, *TP63* gene, *LEPREL1* gene, Enhancer, Genome-wide association study, Single-nucleotide polymorphism

## Abstract

**Purpose:**

Genome-wide association studies (GWAS) have led to the identification of a bladder cancer susceptibility variant (rs710521) in a non-coding intergenic region between the *TP63* and *LEPREL1* genes on chromosome 3q28, suggesting a role in the transcriptional regulation of these genes. In this study, we aimed to functionally characterize the 3q28 bladder cancer risk locus.

**Methods:**

Fine-mapping was performed by focusing on the region surrounding rs710521, and variants were prioritized for further experiments using ENCODE regulatory data. The enhancer activity of the identified region was evaluated using dual-luciferase assays. CRISPR/Cas9-mediated deletion of the enhancer region was performed and the effect of this deletion on cell proliferation and gene expression levels was evaluated using CellTiter-Glo and RT-qPCR, respectively.

**Results:**

Fine-mapping of the GWAS signal region led to the identification of twenty SNPs that showed a stronger association with bladder cancer risk than rs710521. Using publicly available data on regulatory elements and sequences, an enhancer region containing the bladder cancer risk variants was identified. Through reporter assays, we found that the presence of the enhancer region significantly increased *ΔNTP63* promoter activity in bladder cancer-derived cell lines. CRISPR/Cas9-mediated deletion of the enhancer region reduced the viability of bladder cancer cells by decreasing the expression of *ΔNTP63* and p63 target genes.

**Conclusions:**

Taken together, our data show that bladder cancer risk-associated variants on chromosome 3q28 are located in an active enhancer region. Further characterization of the allele-specific activity of the identified enhancer and its target genes may lead to the identification of novel signaling pathways involved in bladder carcinogenesis.

**Electronic supplementary material:**

The online version of this article (10.1007/s13402-018-0393-5) contains supplementary material, which is available to authorized users.

## Introduction

In recent years, bladder cancer risk loci have been identified through genome-wide association studies (GWAS) [1]. Until now, 14 bladder cancer risk loci, including *TP63*, *c-MYC* [2], *TERT/CLPTM1L* [3], *FGFR3/TACC3* [4], *PSCA* [5], *APOBEC3A/CBX6*, *CCNE1*, *UGT1A* [6], *SLC14A1* [7, 8], *JAG1* [9, 10], *TERC*, *LSP1* [10] and *MCF2L* [11] have been described. The GWAS-identified variants are unlikely functional by themselves. Instead, identified single-nucleotide polymorphisms (SNPs) are often correlated to the actual (unmeasured) causal variants, and hence further fine-mapping is required. Moreover, they mostly map to non-coding intronic and intergenic regions [12]. Finding the causative SNPs proved to be challenging and only four of the GWAS-identified bladder risk loci, including *PSCA*, *UGT1A*, *CCNE1* and *APOBEC3B*, have been functionally characterized, showing allele-specific regulation of gene expression [5, 13–16].

The importance of non-coding regions in the regulation of gene expression has been demonstrated by the Encyclopedia of DNA Elements (ENCODE) initiative. ENCODE data revealed that GWAS-identified risk SNPs are significantly enriched in active regulatory regions, which are often cell type and disease specific [17, 18]. The presence of a SNP in a non-coding region can alter gene expression levels both *in cis* (locally) and *in trans* (distally) via multiple mechanisms [19]. For example, one of the most studied colorectal cancer risk SNPs (rs6983267), located in a 8q24 gene desert [20], was found to enhance binding of the TCF4 transcription factor in an enhancer region [21]. This enhancer interacts with the *c-MYC* promoter and modulates its activity in an allele-specific manner [22, 23], leading to increased *c-MYC* expression in the presence of the risk allele [24]. Later it was found that rs6983267 lies within a long non-coding RNA, *CCAT2*, and that colorectal cancer cell lines express significantly higher levels of *CCAT2* transcripts containing the risk G allele. *CCAT2* was found to positively regulate *c-MYC* transcription levels and thereby to affect the growth, metastasis and energy metabolism of colorectal cancer cells in an allele-specific manner [25].

One of the bladder cancer risk SNPs, rs710521 (A > G variant, A is risk-increasing allele) is located in an intergenic region between *TP63* and *LEPREL1* on chromosome 3q28, in a linkage disequilibrium (LD) block encompassing the *TP63* gene [2], which codes for a member of the p53 family of transcription factors. *TP63* encodes two isoforms: *TATP63* and *ΔNTP63,* which have opposite effects on cell cycle regulation and apoptosis. Furthermore, it has been found that p63 expression plays a role in epithelial development and the formation of squamous epithelium. It has also been found to play a role in cancer development [26]. In non-muscle invasive bladder cancer (NMIBC), altered p63 expression has been found to be inversely correlated with pathological grade, whereas in muscle invasive tumors (MIBC) p63 expression has been found to be frequently down-regulated [27]. The second gene within the 3q28 locus, *LEPREL1,* encodes a member of the Leprecan family of proteoglycans, involved in posttranslational modification of collagen, leading to protein stability [28]. The expression of *LEPREL1* has been found to be down-regulated in breast cancer [29] and hepatocellular carcinoma [30] and to be up-regulated in thyroid cancer [31].

In our study, we aimed at fine-mapping the original GWAS signal, rs710521, within the *TP63/LEPREL1* gene locus and, subsequently, at a functional characterization of genetic variants within this locus that are associated with bladder cancer risk. Using publicly available regulatory data an enhancer region containing the fine-mapped bladder cancer risk SNPs was identified. We found that the presence of the identified enhancer increased *ΔNTP63* promoter activity and affected cell viability. From our data we conclude that the bladder cancer-associated SNPs at the 3q28 locus map to an enhancer element that contributes to bladder cancer development by modulating gene expression levels.

## Materials and methods

### Regional association analysis

Fine-mapping was based on association analysis of imputed single nucleotide variant (SNV) data in the 500 kb region surrounding rs710521 in 1601 bladder cancer patients and 1819 controls from the Nijmegen Bladder Cancer Study (NBCS) [8]. The NBCS participants were genotyped using either the Illumina Human370CNV-Duo or the Quad BeadChip and imputed using IMPUTE v2.1 software [32] in conjunction with a training set consisting of the combined 1000 Genomes low-coverage pilot haplotypes (released June 2010, 120 chromosomes) and the HapMap3 haplotypes (released February 2009, 1920 chromosomes). Individuals with < 96% yield for the 292,650 autosomal SNPs present on both chips and SNPs with a minor allele frequency < 0.01, which were not in Hardy-Weinberg equilibrium (*P* < 10–5), or with a different frequency for the two chip types used (*P* < 10–5), were excluded from the imputation. Statistical case-control association analyses for all SNVs were performed using SNPtest (version 2.4) [33] with the most common homozygous genotype as reference genotype.

### Prioritization of variants

The variants that showed a stronger association (in terms of *p*-value) with bladder cancer risk than rs710521 were prioritized for further analysis using the ENCODE data [17, 34], Regulome DB [35] and the Haploreg database [19]. The presence of histone modifications associated with active chromatin, DNaseI hypersensitive sites and binding of transcription factors was used to select potential active regulatory regions [36].

### Cell culture

The bladder cancer-derived cell lines 5637 (ATCC: HTB-9, Manassas, Virginia, USA), 647 V (DSMZ: ACC-414, Braunschweig, Germany), RT-112 (DSMZ: ACC-418), SW800 [37] and T24 (ATCC: HTB-4) were grown in RPMI-1640 medium (Invitrogen, Carlsbad, California, USA) supplemented with 10% fetal calf serum (Sigma-Aldrich, St.Louis, Missouri, USA) and L-glutamine. The cells were cultured in a humidified atmosphere at 37 °C and 5% CO_2_. All cell lines were authenticated in 2016 using the PowerPlex 21 PCR kit (Promega, Madison, Wisconsin, USA) by Eurofins Genomics (Ebersberg, Germany) and tested mycoplasma-free.

### Formaldehyde-assisted isolation of regulatory elements (FAIRE)

FAIRE analysis was performed as described previously [38]. Briefly, 647 V and RT112 bladder cancer cells were seeded in 10-cm culture dishes. When confluent, cells were cross-linked using 37% formaldehyde (Sigma-Aldrich) for 5 min. Subsequently, cross-linked cells were lysed and cell lysates were sonicated on ice using a Bioruptor sonicator (Diagenode, Seraing, Belgium) for 15 min (15 cycles of 30 s. on, 30 s. off). The input control DNA and FAIRE DNA were isolated according to the manufacturer’s protocol. FAIRE enrichment was measured by FAIRE-qPCR using 5 μl (~5 ng/μl) DNA as input, SYBR Green qPCR reagent (Roche) and a LightCycler LC480 instrument (Roche). The relative FAIRE enrichment for each amplicon was calculated as the ratio for the signal from the FAIRE sample relative to the signal from the input control DNA. The primers used are listed in Supplementary Table 1.

### Cloning of enhancer reporter vectors

DNA was isolated from fresh frozen tissue samples (normal bladder adjacent to tumor) using a QIAamp DNA Mini kit (QIAGEN, Hilden, Germany), according to manufacturer’s instructions. The *ΔNTP63* promoter region [39], the *LEPREL1* promoter region and the intergenic enhancer region were PCR-amplified using PrimeSTAR HS DNA polymerase and a GC buffer (Takara, Shiga, Japan). The primer sequences used are listed in Supplementary Table 1. For isolation of the enhancer (E1) containing the risk and the non-risk haplotypes (AA and GG, respectively), the region encompassing rs4687103 and rs4687104 was amplified. Haplotypes were determined using Haploview software [40]. The *ΔNTP63* promoter region and the *LEPREL1* promoter region were ligated into a pGL3-Basic vector (Promega), upstream of the luciferase gene, using *Bgl*II and *Hin*dIII restriction sites (NEB, Ipswich, Massachusetts, USA). Next, the enhancer region was cloned into the pGL3-*ΔNTP63* promoter and the pGL3-*LEPREL1* promoter containing vectors, downstream of the luciferase gene using *Bam*HI and *Sal*I restriction sites (New England Biolabs, NEB). Plasmid DNA was isolated using a QIAGEN Plasmid Midi kit (QIAGEN). All reactions were performed according to the manufacturer’s instructions. The integrity of the vectors was confirmed by Sanger sequencing (Sequencing Facility, Department of Genetics, Radboud university medical center, Nijmegen, The Netherlands).

### Enhancer reporter assays

Bladder cancer-derived cells were seeded in 96-well plates. At 70–80% confluency, the cells were transfected with 900 ng reporter vector and 100 ng pRL-TK control vector (Promega) using X-tremeGENE 9 transfection reagent (Roche), according to manufacturer’s instructions. Two days after transfection, enhancer reporter assays were performed using a Dual-Luciferase reporter assay system (Promega) according to the manufacturer’s instructions. Luciferase activity was measured using a Victor^3^ multilabel reader (PerkinElmer, Waltham, Massachusetts, USA). The *Firefly* luciferase signals were normalized to the *Renilla* luciferase signals. All experiments were performed in triplicate and repeated at least three times. Data were compared to the pGL3-*ΔNTP63* or pGL3-*LEPREL1* promoter activities in each experiment.

### Cloning of CRISPR/Cas9 genome editing vectors

Guide RNAs (gRNAs) targeting the *TP63* enhancer region were designed using the CRISPR Design tool (http://crispr.mit.edu). The gRNAs sequences are listed in Supplementary Table 1. The gRNA cloning vector was a gift from George Church (Addgene plasmid #41824) [41]. The gRNAs and gRNA vector were modified as described before [42]. The oligonucleotides were synthesized (Invitrogen) and annealed according to the gRNA design and cloning protocol from the Church Laboratory (Addgene) using Phusion DNA polymerase (NEB). The gRNA cloning vector was linearized using *Afl*II (NEB) and inserts containing target sequences were incorporated into the gRNA vector using Gibson assembly (NEB), according to manufacturer’s instructions. Cloning products were transformed into DH5α competent *E.coli* (NEB) cells. Plasmid DNA isolation and DNA sequence analyses were performed as described above.

### CRISPR/Cas9-mediated deletion of the *TP63* E1 enhancer region

5637 bladder cancer-derived cells were seeded in 6-well plates. At 70–80% confluency the cells were co-transfected with a hCas9 expression vector (a gift from George Church, Addgene plasmid #41815) [41] and two gRNA vectors (targeting the regions flanking the enhancer), using X-tremeGENE 9 transfection reagent (Roche) according to the manufacturer’s instructions. Three days after transfection, the cells were trypsinized and seeded at low densities in 10-cm dishes. The remaining cells were used for DNA isolation using a QIAamp DNA Mini Kit (QIAGEN) after which deletion-specific PCR was performed using SuperTaq DNA polymerase (ThermoFisher Scientific, Waltham, Massachusetts, USA). Deletion of the 2.2 kb E1 enhancer region was confirmed by agarose gel electrophoresis and Sanger sequencing of the PCR amplicons. The ratio between non-deleted and deleted cells was used to evaluate the efficiency of the CRISPR/Cas9 system (Supplementary Fig. A). After 2 weeks, single cell colonies were harvested and DNA was isolated using a NucleoSpin Tissue XS kit (Macherey-Nagel, Düren, Germany). Deletion-specific PCR was performed (as above) to identify and select E1-deleted clones (see Supplementary Fig. B).

### RNA isolation and RT-qPCR

CRISPR/Cas9 deleted cell lines were seeded in 6-well plates. At 70–80% confluency, total RNA was isolated using TRIzol reagent (Invitrogen). The RNA yield was evaluated using a Nanodrop ND-1000 system (ThermoFisher Scientific). RNA was DNaseI treated, and cDNA was synthesized using random hexamer primers in conjunction with Superscript II reverse transcriptase (Invitrogen). Gene expression was evaluated by SYBR Green qPCR analysis (Roche) on a LightCycler LC480 instrument (Roche). Human Heterochromatin Protein 1, Binding Protein 3 (*HP1BP3*) mRNA expression was used for normalization. Relative gene expression was calculated using the ΔΔCt method, and all data were compared to those of wild-type, non-deleted cells. The primer sequences used are listed in Supplementary Table 1.

### Cell viability assay

Cells were seeded into 96-well plates, after which cell viability was assessed at different time points using a CellTiter-Glo luminescence assay (Promega) according to the manufacturer’s instructions. Luminescence was measured using a Victor^3^ multilabel reader (PerkinElmer). Each experiment was performed in triplicate and repeated at least three times. All data were compared to those of non-deleted cells.

### Correlation of gene expression levels with rs4687103 genotypes

To perform expression quantitative loci (eQTL) analysis, rs4687103 genotype data from 412 MIBC patients were downloaded from The Cancer Genome Atlas (TCGA) project [43]. Only patients from Utah residents of northern and western European ancestry (CEU) were included (*n* = 297). Gene expression data were downloaded from the Broad Institute GDAC Firehose (gdac.broadinstitute.org). From 297 MIBC patients, cases with alterations in the *TP63* and/or *LEPREL1* loci or p63 target genes as shown in cBioPortal [44, 45] were excluded from eQTL analysis (*n* = 27, 35, 23, 31, 15 and 15 for *TP63*, *LEPREL1*, *FGFR3*, *NOTCH1*, *KRT5* and *KR*T6A, respectively). Gene expression levels were compared between genotypes (GG and GA and AA). Clinical data were downloaded from cBioPortal [44, 45].

### Statistical analysis

Statistical analyses of data from the FAIRE assays, the enhancer reporter assays, and the cell proliferation and gene expression assays were performed using GraphPad and a one-way ANOVA test. Associations between survival and rs4687103 genotype were analyzed using IBM SPSS and Kaplan-Meier analysis (log-rank test). For all analyses a *p*-value < 0.05 was considered significant.

## Results

### Association analysis of the rs710521 region

**Fig. 1:**
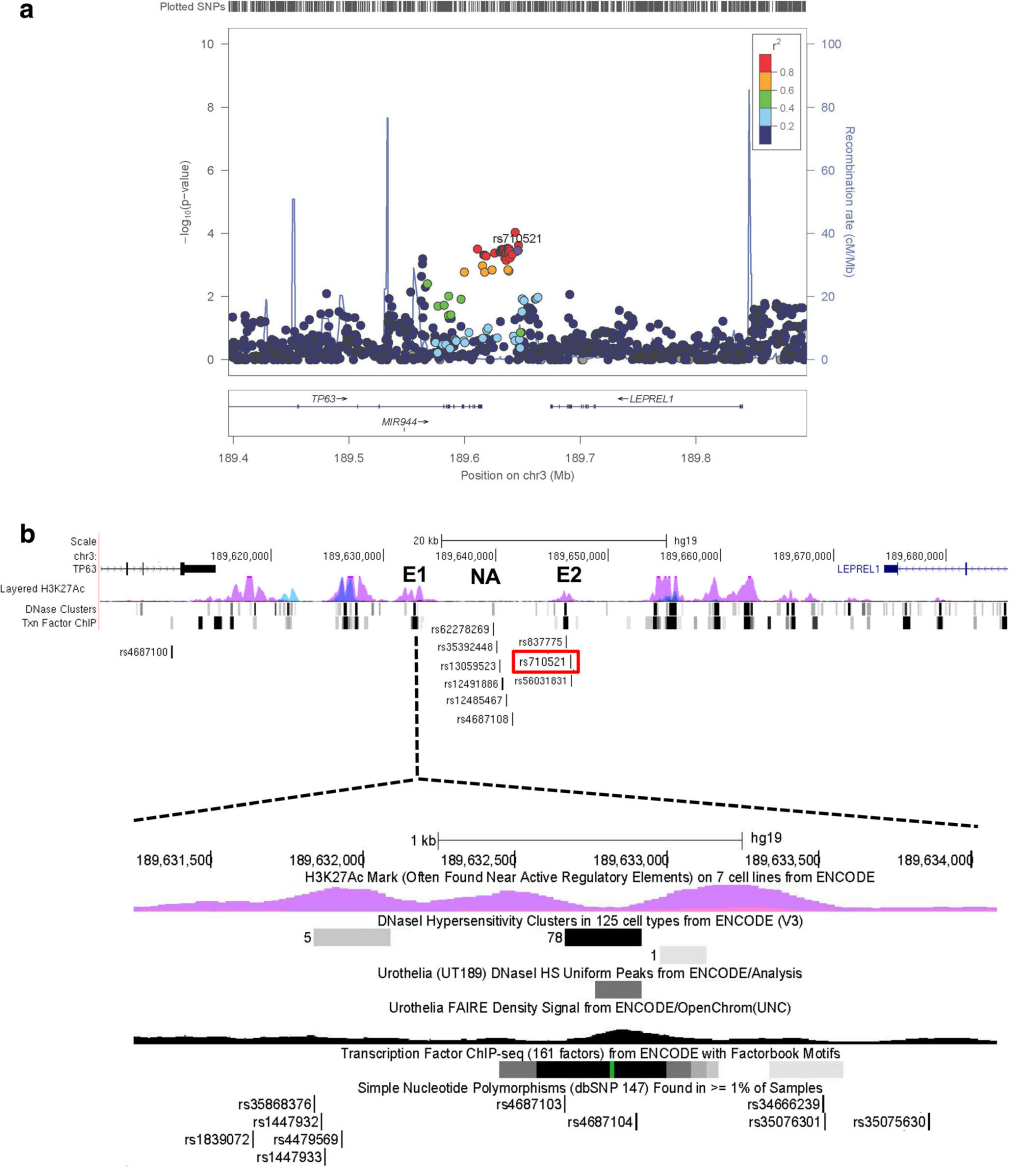
Fine-mapping of the original GWAS hit, rs710521, on 3q28. **a** Regional association plot showing SNPs in 3q28 and their association with bladder cancer risk. In total 20 SNPs are associated with bladder cancer risk (i.e. their *p*-values (y-axis) are equal/lower than those for rs710521 (purple dot) and are in high LD with rs710521 (r^2^ > 0.8). The SNPs are colored based on LD (r^2^) and their genomic positions are based on the hg19 EUR November 2014 version of the reference genome. The plot was created using LocusZoom software [46]. **b** Detailed overview of the *TP63* and *LEPREL1* gene locus, containing the 20 fine-mapped bladder cancer risk SNPs. The identified SNPs cluster in and around two enhancer regions (E1 and E2), which were assigned as enhancers based on the ENCODE regulatory data (i.e., presence of H3K27Ac histone marks, DNaseI hypersensitive sites and transcription factor binding sites). Rs4687100 maps to the last intron of the *TP6*3 gene. Rs1839072, rs35868376, rs1447932, rs1447933, rs4479569, rs4687103, rs4687104, rs34666239, rs35076301 and rs35075630 map to enhancer region 1 (E1). Rs13089435 is located close to E1 in a region were H2K27Ac marks are absent (not shown). Rs62278269, rs35392448, rs13059523, rs12491886, rs12485497 and rs4687108 are located in a non-active region (NA). Rs837775 is located upstream of E2 in the non-active region. The original GWAS SNP, rs710521 (marked in red) and rs56031831 are located in E2. The numbers in the DNase clusters represent the number of ENCODE cell lines in which the region was DNase sensitive. The regulatory data for urothelia show the DNaseI hypersensitive peak and enrichment in the open chromatin structure revealed by FAIRE. Txn: transcription factor binding based on ChIP analysis. The images were downloaded from the UCSC Genome Browser (hg19) [47] and modified

In the analyzed set of bladder cancer patients and controls, the odds ratio (OR) for rs710521 GA versus AA was 0.78 (95% confidence interval 0.68–0.90) and for GG versus AA 0.69 (95% CI 0.52–0.91) with a *p*-value for association (general genotype model) of 0.00034741. There were 20 SNPs that showed a stronger association with reduced bladder cancer risk. These were all in strong linkage disequilibrium (LD; r^2^ > 0.8) with rs710521 and showed similar allele frequencies and ORs (Fig. 1a, Supplementary Table 2).

### In silico analysis of potential regulatory activity of the bladder cancer risk SNPs

Although the interpretation of non-coding GWAS-identified variants is challenging, several characteristics of the DNA regions containing functional variants have been described [19, 36]. In order to prioritize the 21 bladder cancer-associated SNPs within the *TP63* region, we used publicly available regulatory data [19, 34, 35] (Supplementary Table 3). Depending on the database used, 57.2 to 85.7% of the SNPs were found to be located in regions containing histone marks characteristic for enhancers, 19.1 to 33.3% of the SNPs were found to be located in DNaseI hypersensitive sites, and 17 SNPs were predicted to alter transcription factor binding sites. Two SNPs, rs4687103 and rs4687104, were found to be located in multiple transcription factor binding sites (Fig. 1b). Rs4687104 is the only variant that was predicted to alter transcription factor binding motifs (Regulome DB, Supplementary Table 3). Moreover, rs4687104 was found to lie within an active chromatin region in urothelium (marked by a DNaseI hypersensitive, nucleosome-free site) [35], suggesting its potential for regulatory activity. Based on the presence of active histone modifications, DNaseI hypersensitivity, open chromatin structure and the presence of transcription factor binding sites, we selected a putative enhancer region 1 (E1), containing rs1839072, rs35868376, rs1447932, rs1447933, rs4479569, rs4687103, rs4687104, rs34666239, rs35076301 and rs35075630, for further functional studies (Fig. 1b).

### The enhancer 1 region lies within nucleosome-depleted, active chromatin

**Fig. 2:**
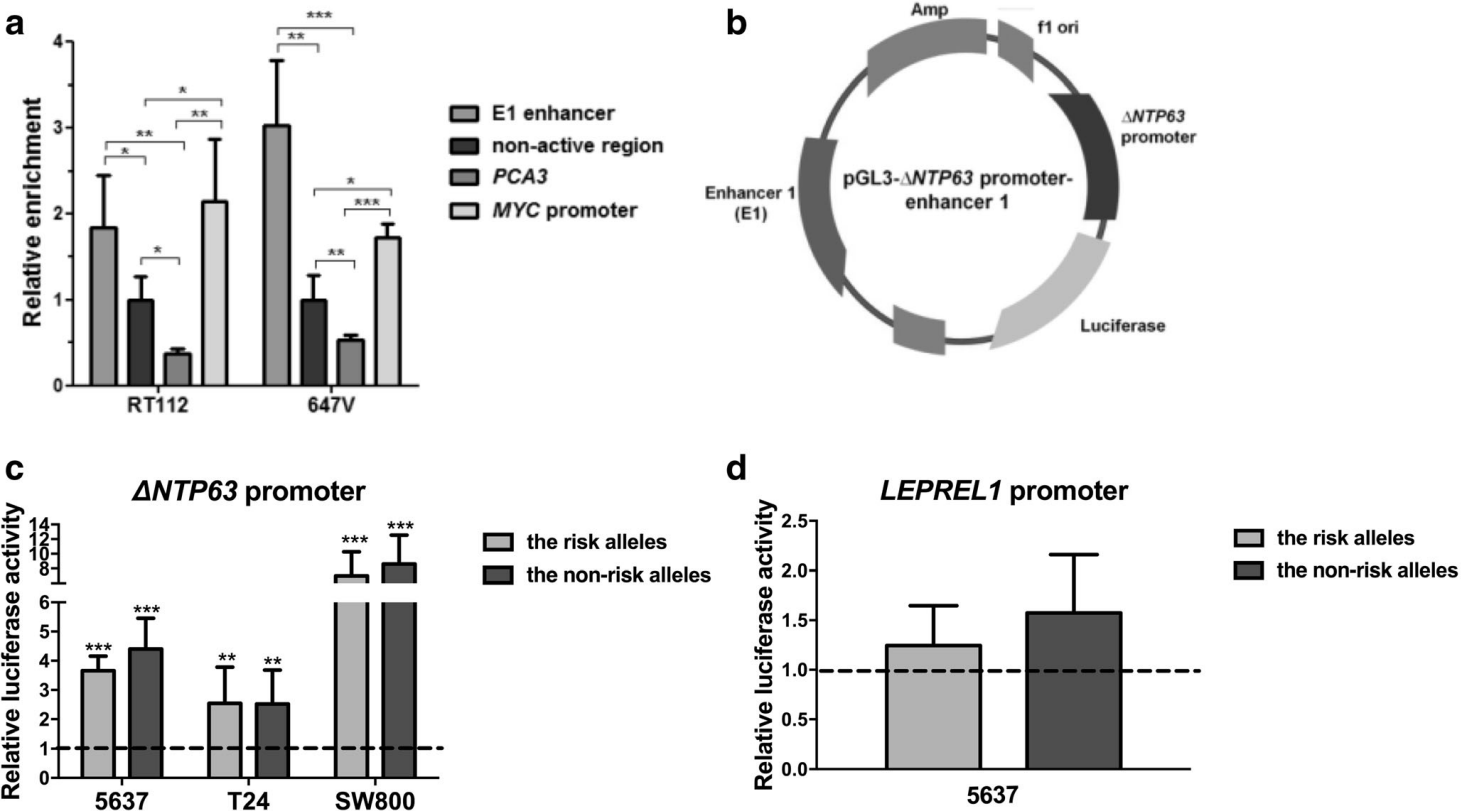
*ΔNTP63/LEPREL1* promoter activity analysis of the E1 enhancer. **a** Enrichment in the open, nucleosome-depleted chromatin in the E1 enhancer region in two bladder cancer-derived cell lines, RT112 and 647 V, analyzed by FAIRE. All data are normalized to a non-active region within the *TP63*/*LEPREL1* locus. **b** Schematic overview of the reporter vector (pGL3-*ΔNTP63* promoter-enhancer) used for promoter-enhancer assays. Enhancer activity in 5637 (*TP63* positive) and T24 and SW800 (*TP63* negative) cells, using the enhancer part containing rs4687104 and rs4687103 with either the minor (non-risk) or the major (risk) alleles, on **c** *ΔNTP63* and **d** *LEPREL1* promoter activity is shown. Data are compared to the pGL3- *ΔNTP63* promoter or the pGL3-*LEPREL1* promoter-only construct, respectively (dashed line). All experiments were performed in triplicate and repeated at least three times using independent plasmid DNA isolations. Bars represent mean ± SD; *p < 0.05; ** *p* < 0.01; *** *p* < 0,001

To confirm that the identified putative enhancer region lies within an active, open chromatin region as shown by the ENCODE data (Fig. 1b), we analyzed its nucleosome occupancy in bladder cancer cells using FAIRE analysis. In two bladder cancer-derived cell lines, RT112 and 647 V, the identified enhancer region E1 showed a significant FAIRE enrichment, confirming nucleosome-depletion and thus an open chromatin structure. The region surrounding rs4687103 in the E1 region showed a higher FAIRE enrichment compared to a non-active heterochromatin region within the *TP63*/*LEPREL1* locus, and compared to the non-expressed prostate-specific *PCA3* gene locus. Additionally, the enrichment in active chromatin in the identified enhancer region was found to be similar to the *c-MYC* promoter region, supporting its potential regulatory activity in bladder cancer cells (Fig. 2a).

### The E1 enhancer region affects *ΔNTP63* and *LEPREL1* promoter activity

Because of the role of enhancer regions in the regulation of gene expression, we hypothesized that the identified enhancer region containing the bladder cancer risk SNPs may (allele-specifically) activate the *ΔNTP63* (the *TP63* isoform that is most abundantly expressed in normal urothelium and bladder cancer, data not shown) and/or *LEPREL1* gene promoter activities *in cis*. We created reporter vectors containing the *ΔNTP63* or *LEPREL1* promoter and the identified E1 enhancer region, the latter containing both the risk and the non-risk haplotypes (Fig. 2b). We found that the presence of the E1 fragment, harboring most of the transcription factor binding sites (containing rs4687103 and rs4687104), significantly increased *ΔNTP63* promoter activity in the presence of both the minor and the major alleles in all three cell line models tested (Fig. 2c). In contrast to the *ΔNTP63* promoter, no regulatory effect of E1 on *LEPREL1* promoter activity was observed (Fig. 2d). Also, no significant allele-specific effects on E1 enhancer activity were observed (Fig. 2c and d).

### Deletion of the E1 enhancer region affects cell viability and *TP63* expression

**Fig. 3:**
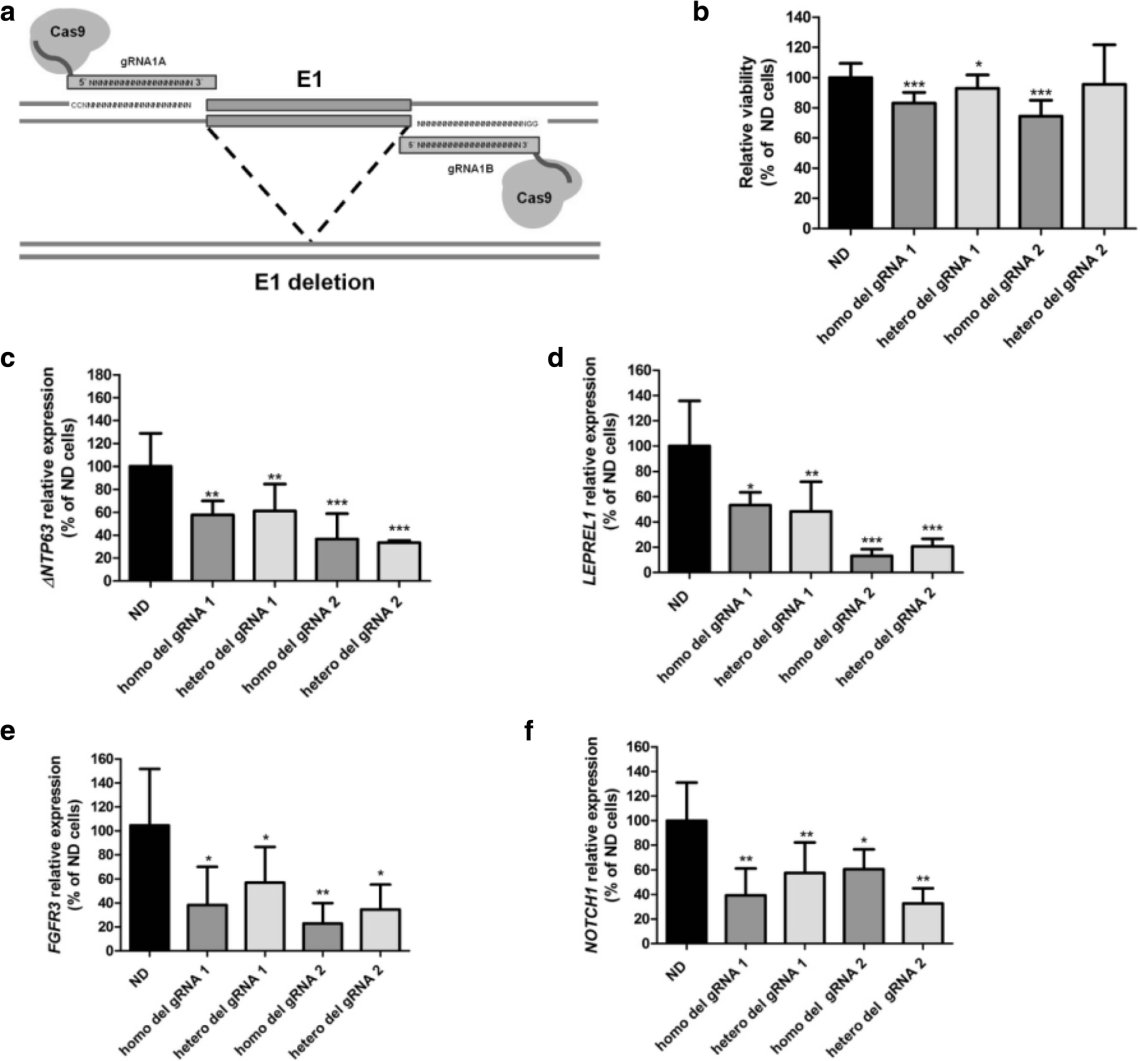
Functional effects of CRISPR/Cas9-mediated deletion of the E1 enhancer region in 5637 bladder cancer-derived cells. **a** Experimental approach used for deletion of enhancer E1 in 5637 cells using a set of gRNAs flanking the identified enhancer region and the wild-type Cas9 protein. The enhancer region was targeted using 2 independent sets of gRNAs (gRNA 1 and gRNA 2). The effects of the deletion on **b** cell viability and **c** *ΔNTP63* and **d** *LEPREL1* gene expression levels are shown, as well as the effect of the deletion on the expression levels of the p63 target genes *FGFR3* (**e**) and *NOTCH1* (**f**). All data from E1-deleted cells from single cell colonies are compared to the average values in wild-type, non-deleted, cells from corresponding experiments. All experiments were performed in triplicate and repeated at least three times. Bars represent mean ± SD; homo, homozygous deletion; hetero, heterozygous deletion; * *p* < 0.05; ** *p* < 0.01; *** *p* < 0.001

We further evaluated the cellular effects of the identified enhancer region by deleting it in 5637 cells using CRISPR/Cas9 technology (Fig. 3a, Supplementary Fig. 1A-B). We found that homozygous deletion of enhancer region E1 significantly decreased cell viability compared to wild-type, non-deleted cells (Fig. 3b). Heterozygous deletion of E1 using gRNA set 1 also led to decreased cell viability compared to non-deleted cells, although to a lesser extent than in homozygous E1 deleted cells (Fig. 3b). Homozygous and heterozygous deletion of E1 affected gene expression *in cis* by decreasing *ΔNTP63* levels (Fig. 3c)*.* Interestingly, expression of the *TP63*-flanking gene *LEPREL1* was found to be decreased in E1-deleted cells as well (Fig. 3d). Additionally, the expression levels of two reported p63 target genes, *FGFR3* [48] and *NOTCH1* [49], were evaluated. *ΔNTP63* expression was found to be positively correlated with *FGFR3* and *NOTCH1* expression in normal urothelium and MIBC tissue samples (Supplementary Fig. 2). Interestingly, the *FGFR3* and *NOTCH1* expression levels were also found to be decreased in E1-deleted cells (Fig. 3e and f, respectively).

### Correlation of gene expression with rs4687103 genotype

**Fig. 4:**
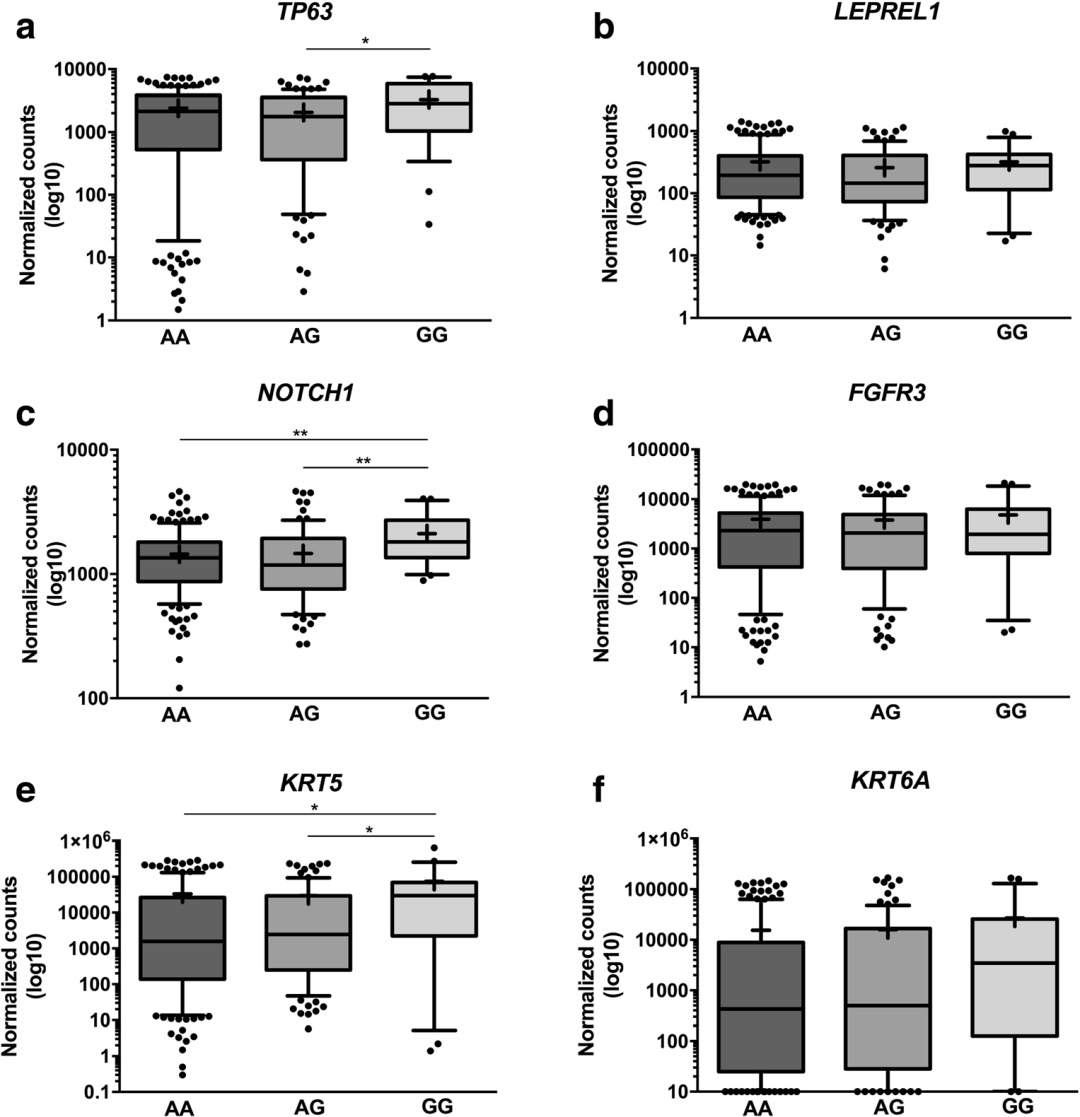
Correlations between rs4687103 genotype and **a** *TP63,* **b** *LEPREL1*, **c** *NOTCH1*, **d** *FGFR3*, **e** *KRT5* and **f** *KRT6A* expression levels in MIBC. The whiskers represent 10th and 90th percentile. The line represents median. + mean; * *p* < 0.05; ** *p* < 0.01

The presence of a SNP may affect gene expression in an allele-specific manner, resulting in differences in gene expression levels between individuals [50]. Concordantly, *TP63* expression was found to be up-regulated in individuals homozygous for the G allele in comparison to heterozygous individuals (Fig. 4a). No significant differences between *LEPREL1* expression levels and rs4687103 genotypes were found (Fig. 4b). The expression levels of one of the p63 target genes, *NOTCH1*, was found to correlate with the rs4687103 genotype. Individuals homozygous for the non-risk G allele exhibited significantly higher *NOTCH1* expression levels compared to carriers of the risk A allele (Fig. 4c). No allele-specific expression of *FGFR3*, another p63 target gene, was found (Fig. 4d). Moreover, the expression levels of *KRT5* (Fig. 4e), but not *KRT6A* (Fig. 4f), were also found to be significantly higher in individuals homozygous for the non-risk G allele. We further evaluated the effect of the rs4687103 genotype on survival using a set of 297 muscle-invasive tumors from the TCGA project [43]. No significant differences in overall survival (OS) were observed (*p* = 0.178, Supplementary Fig. 3A), although a trend towards a shorter OS in MIBC for GG versus GA/AA genotypes was found (*p* = 0.065, Supplementary Fig. 3B).

## Discussion

Susceptibility to bladder cancer has been shown to be modified by complex interactions between genetic and environmental factors [51]. Several genetic variants have been identified that are associated with bladder cancer risk, which point to novel genes and mechanisms involved in bladder cancer development [12]. Here, we fine-mapped a GWAS signal at the 3q28 locus and showed that a region containing the bladder cancer-associated SNPs regulate gene expression levels and modulate cell survival.

Using publicly available data for regulatory elements, we identified an enhancer region that significantly increased *ΔNTP63* promoter activity in bladder cancer-derived cells, although not in an allele-specific manner. Genetic variants identified by GWAS are usually associated with small risks and their effects on gene expression levels may be modest [12]. Therefore, the sensitivity of the current assays may not be sufficient to reveal allele-specific effects. Furthermore, an enhancer region may regulate multiple target genes and a single gene may be regulated by multiple enhancer elements. CRISPR/Cas9 genome editing allows for assessment of the impact of a single genetic variant within particular genomic and/or cell type contexts [52]. Improvements in the efficiency of CRISPR/Cas9-mediated homology directed repair (HDR) [53] may facilitate functional studies of GWAS-identified variants in the future. The expression of transcription factors is tightly controlled and hence no significant, allele-specific effect on the promoter activity and expression of transcription factors (TFs) may be observed. However, allele-specific differences could be manifested through expression of TF-target genes [54]. We indeed found that the expression levels of the *ΔNTP63* target gene *NOTCH1* significantly correlated with the risk SNP genotype. The Notch signaling pathway has been shown to be involved in bladder cancer development, although it is not clear yet whether Notch signaling is oncogenic or tumor suppressive. For example, high expression of Jagged2 (a NOTCH ligand) has been shown to be associated with tumor aggressiveness and the formation of metastases [55]. Other studies have shown that *NOTCH* inactivating mutations in vivo accelerated the development of bladder cancer and promoted the formation of squamous cell carcinoma [56, 57].

In MIBC tissue specimens, the levels of *ΔNTP63* do correlate with the risk SNP genotype. Previously, it was found that in response to genotoxic stress ΔN-p63 is recruited to a p53-binding element in its own promoter leading to silencing of *ΔNTP63* expression [58]. Smoking is the most important risk factor for developing bladder cancer, accounting for 50% of the tumors [51]. Therefore, it is plausible that the modest allele-specific differences in *ΔNTP63* expression are manifested upon (smoking-induced) DNA damage. Moreover, eQTL discovery in tumor tissue has been shown to be challenging due to frequent genetic and epigenetic alterations affecting transcript levels [54].

In our study, the deletion of the enhancer region in the 5637 bladder cancer-derived cell line led to decreases in *ΔNTP63* and p63 target gene mRNA expression levels, subsequently affecting cell viability. Similarly, it has previously been shown that shRNA-mediated silencing of *ΔNTP63* expression in 5637 bladder cancer cells resulted in cell cycle arrest, a decreased proliferation rate and Cyclin D1 downregulation. Moreover, it has been reported that also in a 5637 xenograft model in mice, knockdown of *ΔNTP63* inhibited tumor growth through induction of apoptosis [59]. In another study, knockdown of *ΔNTP63* was found to increase adhesion and to decrease migration of 5637 cells by increasing F-actin levels [60]. Interestingly, reduced *ΔNTP63* expression has been found to sensitize 5637 bladder cancer cells to DNA damage-induced apoptosis independent of p53 [61]. In contrast, Fukushima et al. showed that *ΔNTP63* knockdown led to upregulation of N-cadherin and increased motility and invasion of 5637 cells [62].

We found that deletion of the identified enhancer region E1 led to a decrease in both *ΔNTP63* and *LEPREL1* expression levels. In thyroid carcinoma, *LEPREL1* has been found to serve as a target of the TWIST1 transcription factor (associated with epithelial-to-mesenchymal transition, metastasis formation and a poor prognosis). In addition, it has been reported that down-regulation of *LEPREL1* significantly reduced the growth of thyroid cancer cells [31]. In hepatocellular and breast cancer overexpression of *LEPREL1* has been found to inhibit cell proliferation and colony formation [29, 30]. Until now, however, the role of *LEPREL1* has not been studied in bladder cancer.

Luminal and basal subtypes of MIBC have been identified by several groups [43, 63–66]. The basal subtype has been shown to be associated with a shorter overall survival and to be characterized by deregulation of p63 target genes [65]. High expression of basal keratins, such as KRT5 and KRT6A, is the most characteristic feature of the basal subtype of bladder cancer [63]. ΔNp63 has been shown to regulate *KRT5* and *KRT6A* gene expression in several tissues [65, 67, 68]. The rs4687103 non-risk allele was found to be significantly correlated with increased *KRT5*, but not *KRT6A*, expression, suggesting that the risk locus on 3q28 stimulates basal/squamous differentiation of the tumors, with a shorter overall survival.

Multiple transcription factor binding sites have been shown to overlap the rs4687103 and rs4687104 SNPs in the *TP63* E1 enhancer region, including proteins known to be involved in basal/squamous bladder cancer subtype development, like STAT3 and c-MYC [65, 66]. Moreover, STAT3, FOS, c-MYC and CEBPB transcription factors have been found to bind to the promoter regions of the *KRT5* and *KRT6A* genes [66]. Further studies are required to evaluate which transcription factors can bind to the E1 enhancer in an allele-specific manner, both in normal urothelium and in bladder cancer cells.

Genome-wide association studies have successfully led to the identification of genetic variants that modulate gene expression levels in bladder cancer. The presence of rs2294008, a missense variant in the *PSCA* gene, has been found to lead to formation of a truncated PSCA protein, due to alteration of the start codon. The presence of the variant allele has been found to be associated with reduced *PSCA* promoter activity [5]. The protective T allele of rs17863783 within the *UGT1A* locus has been found to be associated with increased *UGT1A6.1* expression, leading to a higher clearance of carcinogens from the urothelium and a decreased bladder cancer risk [15]. In addition, urine concentration was found to be significantly decreased in carriers of the risk T allele of rs10775480 (intron of *SLC14A1*) [69]. In the *CCNE1* locus, the original GWAS-identified SNP rs8102137 and a *CCNE1* promoter variant rs7257330 were found to be associated with bladder cancer aggressiveness, marked by increased CCNE1 protein expression [14]. *APOBEC3B* expression and enrichment in *APOBEC3B*-signature mutations have been shown to correlate with rs1014971. Moreover, the presence of a SNP within the non-coding region upstream of *APOBEC3A* has been found to affect binding of proteins in an allele-specific manner [16]. Recently, Wang et al. identified a bladder cancer risk variant within the 3’-UTR of the *TP63* gene. The presence of this variant was shown to disrupt *miR-140-5p* binding leading to allele-specific TP63 expression [70]. Additional functional studies are warranted to uncover the molecular mechanisms by which this and other GWAS-identified SNPs influence bladder cancer risk.

In conclusion, we identified an enhancer region within the *TP63*/*LEPREL1* intergenic locus containing bladder cancer risk SNPs that regulate gene expression levels *in cis* and, subsequently, tumor cell viability. Our study underlines the importance of GWAS-identified signals in non-coding regions for bladder cancer development. Further characterization of the identified region may unravel novel allele-specific pathways involved in the modulation of bladder cancer susceptibility.

## Electronic supplementary material

ESM 1 (DOCX 502 kb)

ESM 2 (DOCX 131 kb)
