# Supplementary material for: Identification of an enhancer region within the TP63/LEPREL1 locus containing genetic variants associated with bladder cancer risk
Source: Cell Oncol (Dordr). 2018 Jun 28;41(5):555–68. doi: 10.1007/s13402-018-0393-5 (PMC6153957; doi:10.1007/s13402-018-0393-5)

**SUPPLEMENTARY FIGURES CELLULAR ONCOLOGY**

**Identification of an enhancer region in the TP63/LEPREL1 locus containing genetic variants associated with bladder cancer risk.**

Aleksandra M. Dudek^1^, Sita H. Vermeulen^2^, Dimitar Kolev^2^, Anne J. Grotenhuis^2^, Lambertus A.L.M. Kiemeney ^1,2^, Gerald W. Verhaegh^1,*^

^1^ Radboud university medical center, Radboud Institute for Molecular Life Sciences, Department of Urology, Nijmegen, The Netherlands,

^2^ Radboud university medical center, Radboud Institute for Health Sciences, Department for Health Evidence, Nijmegen, The Netherlands.

*, Gerald W. Verhaegh, Geert Grooteplein Zuid 28, 6525 GA Nijmegen, the Netherlands. Phone: +31243610510. email: Gerald.Verhaegh@radboudumc.nl

**Supplementary Fig 1.** Example of the screening of the CRISPR-Cas9-deleted cells using deletion-specific PCR and agarose gel electrophoresis in A) a pool of E1-deleted cells and B) E1-deleted single cell colonies. Two sets of primer pairs were used to evaluate the presence of the enhancer (E1) deletion. Deletion-specific primer pair (D) was designed in the regions flanking the deleted enhancer, resulting in formation of a short product in the presence of a deletion. Control primer pair (C) was designed within the E1 region. resulting in formation of a short product if the enhancer region was not deleted. The cell line was assigned as negative (PCR product detected only using control primer pair), heterozygous (PCR product detected using control and deletion-specific primer pair) or homozygous (PCR product detected only using deletion-specific primer pair). NT-non-transfected cells. D- CRISPR/Cas9-deleted cells. ND- non-deleted cells; M- 100bp DNA ladder. Grey box indicates the E1 deletion in CRISPR/Cas9-transfected cells.


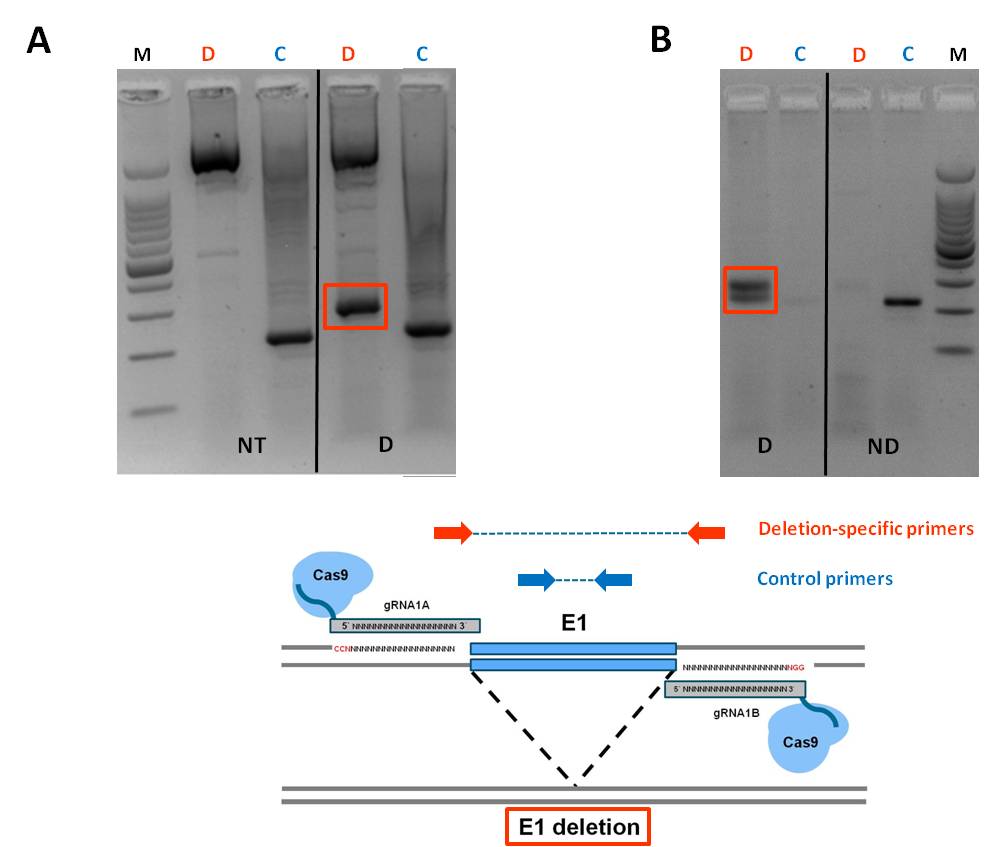


**Supplementary Fig 2.** Correlation between *ΔNTP63* and p63 target genes: *FGFR3* and *NOTCH1* expression levels in (A, C) normal urothelium and (B, D) MIBC using TCGA data (Network TCGA 2014).


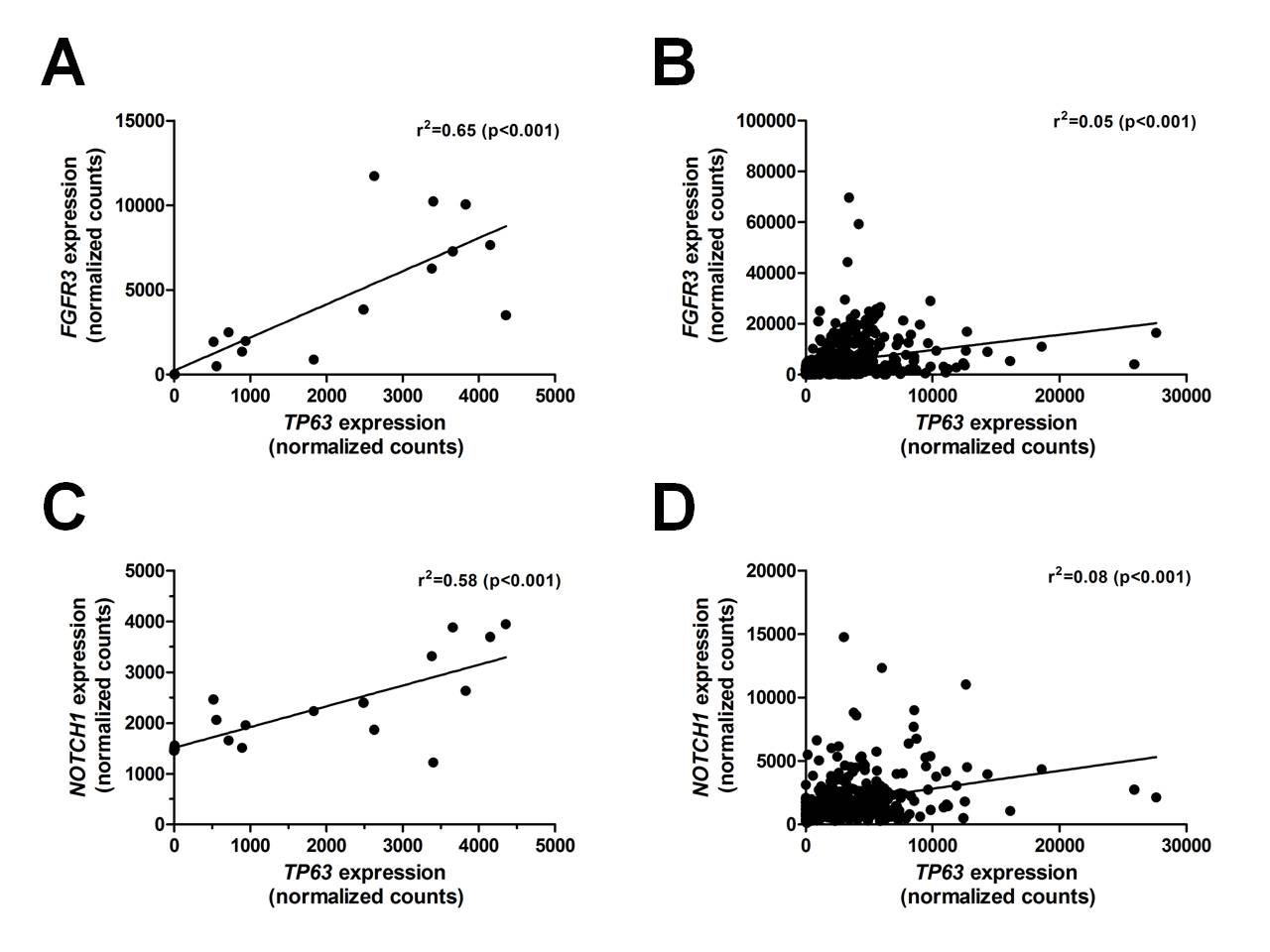


**Supplementary Fig 3.** The effect of rs4687103 on overall survival of MIBC patients from TCGA (Network TCGA 2014) (**A**) stratified by rs4687103 genotype (GG, n=26; GA, n=99; AA, n=170) and (**B**) stratified by rs4687103 genotype using GG and GA/AA genotypes.


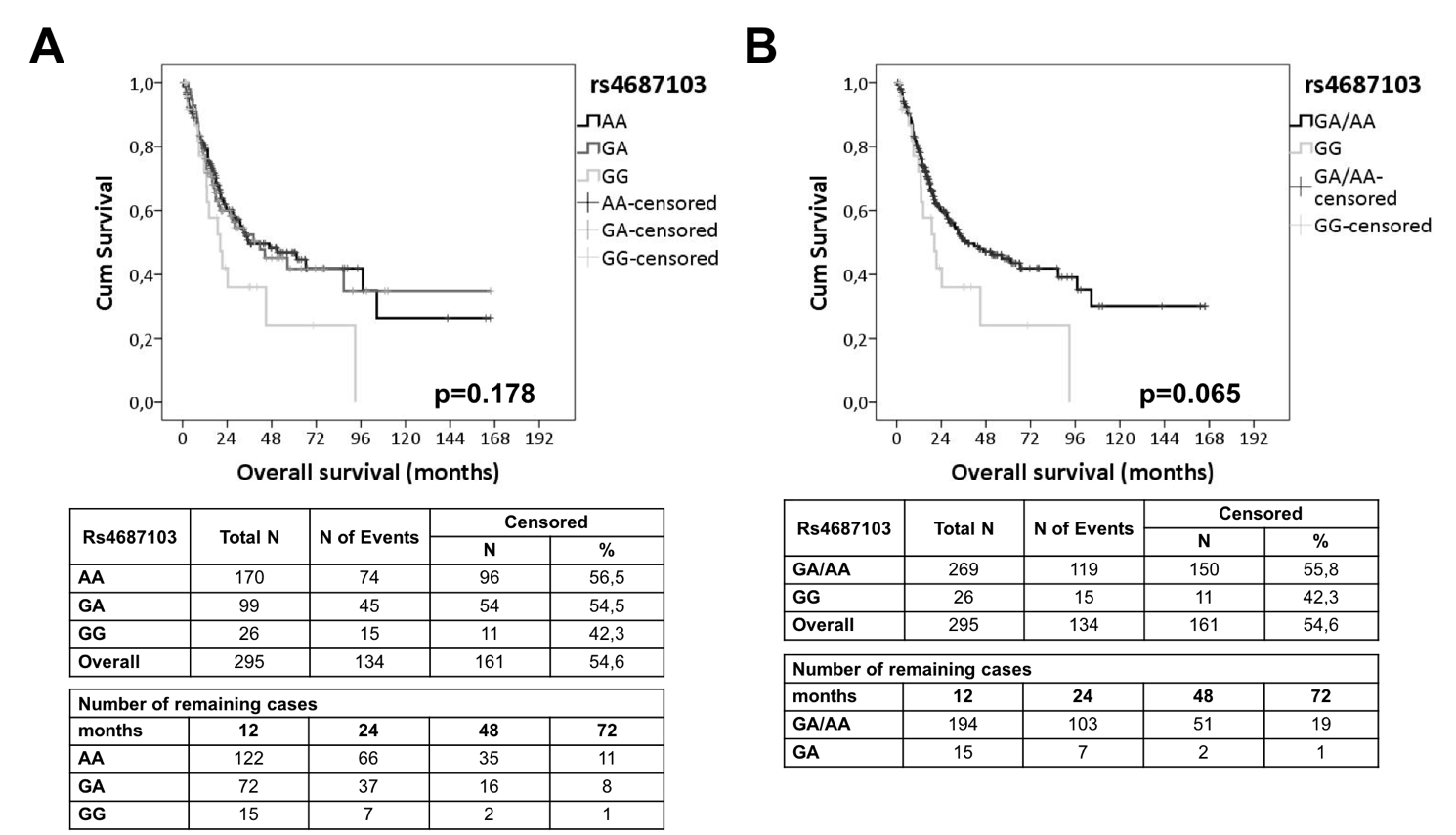

Supplement: Supplementary file 1 — (DOCX 502 kb) [file 13402_2018_393_MOESM1_ESM.docx]
