# Supplementary material for: Identification of an enhancer region within the TP63/LEPREL1 locus containing genetic variants associated with bladder cancer risk
Source: Cell Oncol (Dordr). 2018 Jun 28;41(5):555–68. doi: 10.1007/s13402-018-0393-5 (PMC6153957; doi:10.1007/s13402-018-0393-5)
Supplement: Supplementary file 2 — (DOCX 131 kb) [file 13402_2018_393_MOESM2_ESM.docx]

**SUPPLEMENTARY TABLES CELLULAR ONCOLOGY**

**Identification of an enhancer region in the TP63/LEPREL1 locus containing genetic variants associated with bladder cancer risk.**

Aleksandra M. Dudek^1^, Sita H. Vermeulen^2^, Dimitar Kolev^2^, Anne J. Grotenhuis^2^, Lambertus A.L.M. Kiemeney ^1,2^, Gerald W. Verhaegh^1,*^

^1^ Radboud university medical center, Radboud Institute for Molecular Life Sciences, Department of Urology, Nijmegen, The Netherlands,

^2^ Radboud university medical center, Radboud Institute for Health Sciences, Department for Health Evidence, Nijmegen, The Netherlands.

*, Gerald W. Verhaegh, Geert Grooteplein Zuid 28, 6525 GA Nijmegen, the Netherlands. Phone: +31243610510. email: Gerald.Verhaegh@radboudumc.nl

**Supplementary Table 1.** Primers and gRNA target sequences used for cloning.

| **Primer** | **Forward primer**  **(5`🡪3`)** | | **Reverse primer**  **(5`🡪3`)** | |
| --- | --- | --- | --- | --- |
| **Primers cloning** | | | | |
| ***ΔNTP63* promoter** | TGTGAGGATACCTTAAAGCAAAAA | | TGTTAGCTGTAAGATTGATCAATGC | |
| ***LEPREL1* promoter** | CTACAGGGCCTCTATGGGTTTT | | GGTCCCCTCTCCCACCTT | |
| **Enhancer** | TTTTGTTTTTGACAGGGTCTTG | | TTCCCCTTTTGGTCATTTTG | |
| **Primers RT-qPCR** | | | | |
| ***HP1BP3*** | TGGAATATGCAATCTTGTCTGC | | GAACCCTTTCCCAGAGATCTG | |
| ***TATP63*** | TTCTGGAACAGCCTATATGTTCAGTT | | TCTTCTGATGGTTCATCCACAAA | |
| ***ΔNTP63*** | CAATGCCCAGACTCAATTTAGTGA | | TGCTGGTCCATGCTGTTCAG | |
| ***FGFR3*** | ACGTTACCGTGCTCAAGTCC | | AGCTCCTCCTCGGCTGCT | |
| ***NOTCH1*** | CACTGTGGGCGGGTCC | | GTTGTATTGGTTCGGCACCAT | |
| **Primers FAIRE** | | | | |
| **Enhancer**  (rs4687103) | AAGGCAAACAATTTTTCAATCA | | TCTTTTTGTGTGTGCCTGTTAGA | |
| **Non-active region**  (rs4687108) | CAAATGGTTTCATGACGGATT | | GCTGAGGTGTTTGTTTTGCTT | |
| ***MYC* promoter** | CAATGCGTTGCTGGGTTAT | | CAGAGCGTGGGATGTTAGTG | |
| ***PCA3*** | GCTTCTCGGCACTTGCTATT | | TGGTTGTATGTGGGTTGGC | |
| **CRISPR/Cas9 editing** | | | | |
| **gRNA oligos** | **Targeted region (5`🡪3`)** | | **Selected target sequence for gRNA cloning** | |
| **Enhancer gRNA set 1** |  | |  | |
| gRNA 1.1A | TATTAGTAGATGGTTGTTTAAGG | | GATTAGTAGATGGTTGTTTA | |
| gRNA 1.1B | AGTCTCTAAGTAGGCCTATAAGG | | GAGTCTCTAAGTAGGCCTATA | |
| **Enhancer gRNA set 2** |  | |  | |
| gRNA 1.2A | CATGGTATTAGTTATCCTCCTGG | | GATGGTATTAGTTATCCTCC | |
| gRNA 1.2B | TTTGATGCCCCAAATAATGTAGG | | GTTGATGCCCCAAATAATGT | |
| **E1 (deletion)-specific primers** | | | | |
| **Primer** | | **Forward primer**  **(5`🡪3`)** | | **Reverse primer**  **(5`🡪3`)** |
| **E1 1 (deletion-spec.)** | GTGCCACCACACTAGGCTCT | | TGACCTTGACCTTGTTTCTTAGTTT | |
| **E1 2 (control)** | TGTATGCCACCCACTTCTGA | | TGAGCAAAATACAGGCACCA | |

Red, PAM sequence; green, first nucleotide of gRNA sequence changed into G for U6 promoter-driven expression.

**Supplementary Table 2.** Fine-mapping of the rs710521 at the 3q28 locus. Table shows all SNPs associated with bladder cancer risk with p value lower than for original GWAS hit- rs710521 and in high LD with rs710521 (r^2^>0.8).

| **SNP** | **Bp position**  **(hg19)** | **Major**  **Allele** | **Minor**  **Allele** | **MAF**  **patients** | **MAF**  **controls** | **Heterozygous^^^**  **OR**  **(95% CI)** | **Homozygous^^^**  **OR**  **(95% CI)** | **p-value** | **Imputation score^$^** | | **HWE**  **p-value ^#^** |
| --- | --- | --- | --- | --- | --- | --- | --- | --- | --- | --- | --- |
| rs4687100 | 189611026 | A | G | 0,240 | 0,281 | 0.77 (0.67-0.90) | 0.70 (0.53-0.93) | 0,000308 | 0,9867 | 0,1824 | |
| rs1839072 | 189631390 | A | G | 0,244 | 0,285 | 0.77 (0.67-0.89) | 0.70 (0.53-0.93) | 0,000323 | 0,9850 | 0,2053 | |
| rs35868376 | 189631591 | G | A | 0,244 | 0,285 | 0.77 (0.67-0.89) | 0.70 (0.53-0.93) | 0,000321 | 0,9851 | 0,2053 | |
| rs1447932 | 189631615 | A | G | 0,243 | 0,285 | 0.77 (0.67-0.89) | 0.70 (0.53-0.93) | 0,000321 | 0,9852 | 0,2053 | |
| rs1447933 | 189631627 | A | C | 0,243 | 0,285 | 0.77 (0.67-0.89) | 0.70 (0.53-0.93) | 0,000321 | 0,9852 | 0,2053 | |
| rs4479569 | 189631682 | C | T | 0,243 | 0,285 | 0.77 (0.67-0.89) | 0.70 (0.53-0.93) | 0,000321 | 0,9852 | 0,2053 | |
| rs4687103 | 189632414 | A | G | 0,243 | 0,285 | 0.77 (0.67-0.89) | 0.70 (0.53-0.93) | 0,000318 | 0,9856 | 0,2053 | |
| rs4687104 | 189632650 | A | G | 0,243 | 0,285 | 0.77 (0.67-0.89) | 0.70 (0.53-0.93) | 0,000317 | 0,9857 | 0,2053 | |
| rs34666239 | 189633236 | T | A | 0,230 | 0,270 | 0.78 (0.68-0.90) | 0.68 (0.51-0.92) | 0,000322 | 0,9593 | 0,0742 | |
| rs35076301 | 189633270 | T | C | 0,243 | 0,285 | 0.77 (0.67-0.89) | 0.70 (0.53-0.93) | 0,000315 | 0,9858 | 0,2053 | |
| rs35075630 | 189633612 | G | A | 0,243 | 0,285 | 0.77 (0.67-0.89) | 0.70 (0.53-0.93) | 0,000316 | 0,9858 | 0,2053 | |
| rs13089435 | 189634669 | G | T | 0,243 | 0,285 | 0.77 (0.67-0.89) | 0.70 (0.53-0.93) | 0,000314 | 0,9859 | 0,2053 | |
| rs62278269 | 189636720 | G | T | 0,238 | 0,279 | 0.78 (0.67-0.89) | 0.69 (0.52-0.92) | 0,000290 | 0,9717 | 0,1806 | |
| rs35392448 | 189636753 | A | G | 0,243 | 0,285 | 0.77 (0.67-0.89) | 0.70 (0.53-0.93) | 0,000313 | 0,9862 | 0,2053 | |
| rs13059523 | 189636944 | G | A | 0,243 | 0,285 | 0.77 (0.67-0.89) | 0.70 (0.53-0.93) | 0,000313 | 0,9862 | 0,2053 | |
| rs12491886 | 189637337 | C | T | 0,243 | 0,285 | 0.77 (0.67-0.89) | 0.70 (0.53-0.93) | 0,000313 | 0,9862 | 0,2053 | |
| rs12485467 | 189637494 | A | G | 0,243 | 0,285 | 0.77 (0.67-0.89) | 0.70 (0.53-0.93) | 0,000312 | 0,9862 | 0,2053 | |
| rs4687108 | 189638237 | A | G | 0,243 | 0,285 | 0.77 (0.67-0.89) | 0.70 (0.53-0.93) | 0,000312 | 0,9863 | 0,2053 | |
| rs837775 | 189643423 | C | T | 0,229 | 0,271 | 0.75 (0.65-0.87) | 0.72 (0.54-0.96) | 0,000091 | 0,9530 | 0,2587 | |
| **rs710521**^&^ | **189645683** | **A** | **G** | **0,241** | **0,283** | **0.78 (0.68-0.90)** | **0.69 (0.52-0.91)** | **0,000347** | **1** | **0,2476** | |
| rs56031831 | 189646266 | T | C | 0,236 | 0,278 | 0.78 (0.68-0.90) | 0.67 (0.51-0.90) | 0,000235 | 0,9646 | 0,2663 | |

^, Heterozygous OR indicates risk for heterozygous genotype versus most common homozygous genotype, homozygous OR indicates risk for least common homozygous genotype versus most common homozygous genotype; ^$^, Imputation score is the SNPtest info score (note that all SNPs except rs710521 are imputed); ^#^, HWE test was performed in controls only; ^&^, genotype distribution for rs710521 in patients: 918 AA, 586 AG, 92 GG; in controls: 925 AA, 759 AG, 135 GG. MAF- minor allele frequency; OR-odds ratio; 95% CI- 95% confidence intervals.

**Supplementary Table 3.** Evaluation of 21 identified SNPs associated with bladder cancer risk using publicly available functional data (*in silico* and experimental).

| **SNP** | **HaploReg** | | | **ENCODE (UCSC Genome Browser)** | | | |
| --- | --- | --- | --- | --- | --- | --- | --- |
|  | **Histone marks** | **DNaseI** | **Motif change**  **/protein bound** | **Chromatin state** | **DNaseI** | **TF binding** | **H3K27Ac** |
| rs4687100  (intronic) | no | no | no | weak transcription,  transcription elongation | no | no | no |
| rs1839072 | yes (enhancer 5 tissues) | no | 5 altered motifs | enhancer | no | no | yes |
| rs35868376 | yes (enhancer 7 tissues) | no | 12 altered motifs | enhancer | yes | no | yes |
| rs1447932 | yes (enhancer 7 tissues) | no | 3 altered motifs | enhancer | yes | no | yes |
| rs1447933 | yes (enhancer 7 tissues) | no | no | enhancer | yes | no | yes |
| rs4479569 | yes (enhancer 7 tissues) | no | 4 altered motifs | enhancer/insulator | yes | no | yes |
| rs4687103^*^ | yes (promoter 1 tissue. enhancer 10 tissues) | yes | no | enhancer | yes | multiple | yes |
| rs4687104^*,**^ | yes (promoter 3 tissues. enhancer 9 tissues) | yes | 9 altered motifs | enhancer | yes | multiple | yes |
| rs34666239 | yes (enhancer 8 tissues) | no | 22 altered motifs | enhancer | no | FAM48A | yes |
| rs35076301 | yes (enhancer 8 tissues) | no | 19 altered motifs | enhancer | no | FAM48A | yes |
| rs35075630 | yes (enhancer 3 tissues) | no | 2 altered motifs | enhancer | no | no | yes |
| rs13089435 | yes (enhancer 1 tissue) | no | 2 altered motifs | heterochromatin | no | no | no |
| rs62278269 | yes (enhancer 2 tissues) | no | MYC altered motif | heterochromatin | no | no | no |
| rs35392448 | yes (enhancer 3 tissues) | no | 17 altered motifs | heterochromatin | no | no | no |
| rs13059523 | yes (enhancer 22 tissues) | no | IRX altered motif | heterochromatin | no | no | no |
| rs12491886 | yes (enhancer 3 tissues) | no | 8 altered motifs | heterochromatin | no | no | no |
| rs12485467 | yes (enhancer 2 tissues) | no | p300 altered motif | heterochromatin | no | no | no |
| rs4687108 | no | no | 4 altered motifs | heterochromatin | no | no | no |
| rs837775 | no | no | no | weak transcription | yes | no | no |
| rs710521 | yes (enhancer 3 tissues) | yes | 3 altered motifs | enhancer | no | TFAP2C | yes |
| rs56031831 | yes (enhancer 7 tissues) | yes | 3 altered motifs | enhancer | no | no | yes |

*SiPhy Cons (Haploreg) evolutionary conserved; ** Regulome DB database^35^, the only SNP likely to affect binding of transcription factors and lies in DNaseI hypersensitive site in urothelium; Histone marks and DNaseI hypersensitivity data in HaploReg database are retrieved from the Roadmap Epigenomics Consortium; TF-transcription factor.
